# Increased risk of cardiovascular events and death in the initial phase after discontinuation of febuxostat or allopurinol: another story of the CARES trial

**DOI:** 10.1136/rmdopen-2021-001944

**Published:** 2022-06-22

**Authors:** Byeong-zu Ghang, Ji Sung Lee, Jihye Choi, Jinseok Kim, Bin Yoo

**Affiliations:** 1 Rheumatology, Jeju National University College of Medicine and Graduate School of Medicine, Jeju National University Hospital, Jeju, The Republic of Korea; 2 Department of Clinical Epidemiology and Biostatistics, Asan Medical Center, University of Ulsan College of Medicine, Seoul, The Republic of Korea; 3 Clinical Research Center, Asan Institute for Life Sciences, Asan Medical Center, Seoul, The Republic of Korea; 4 Rheumatology, Asan Medical Center, University of Ulsan College of Medicine, Seoul, The Republic of Korea

**Keywords:** Atherosclerosis, Cardiovascular Diseases, Gout

## Abstract

**Objectives:**

The Cardiovascular Safety of Febuxostat or Allopurinol in Patients with Gout (CARES) trial suggested a higher risk of cardiovascular (CV) death from febuxostat than from allopurinol. However, a significant number of patients died after discontinuation of febuxostat or allopurinol. We investigated whether major adverse cardiovascular events (MACE) and CV death were increased because of discontinuation of febuxostat or allopurinol using the CARES trial data.

**Methods:**

We compared the MACE that occurred during administration and after discontinuation in the initial phase after discontinuation, and we compared the CV and non-CV mortality rates in the initial phase after discontinuation to determine the impact of discontinuation of febuxostat or allopurinol.

**Results:**

Among 6190 patients, the incidence rate per 100 person-years for MACE was 3.11 during administration and 6.71 after discontinuation. MACE was significantly increased after discontinuation compared with that during administration within 1 month (HR 7.40; 95% CI 5.38 to 10.17) and 6 months (HR 5.22; 95% CI 4.26 to 6.39). In the analysis excluding death induced by adverse events that occurred up to 1 day after the last medication, the CV mortality rate was higher than the non-CV mortality rate within 6 months (45.7% vs 27.9%, p=0.0001). In addition, changes in serum uric acid levels from baseline to the last measurement before discontinuation were significantly associated with higher MACE risk after drug discontinuation (HR 1.14; 95% CI 1.04 to 1.26).

**Conclusions:**

MACE and CV death were increased in the initial stage after discontinuation of febuxostat or allopurinol in patients with gout.

**WHAT IS ALREADY KNOWN ON THIS TOPIC**

- The Cardiovascular Safety of Febuxostat or Allopurinol in Patients with Gout (CARES) trial suggested a higher risk of cardiovascular (CV) death from febuxostat than from allopurinol.
- However, a significant number of patients died after discontinuation of febuxostat or allopurinol.

**WHAT THIS STUDY ADDS**

- In patients with gout and major coexisting CV conditions, major adverse cardiovascular event (MACE) and CV death were increased in the initial stage after discontinuation of febuxostat or allopurinol.
- Changes in serum uric acid levels from baseline to last measurement before discontinuation was significantly associated with higher MACE risk after drug discontinuation.

**HOW THIS STUDY MIGHT AFFECT RESEARCH, PRACTICE AND/OR POLICY**

- The results of the CARES trial actually indicate that discontinuing febuxostat or allopurinol may cause more CV events and death than continuing febuxostat or allopurinol, associated with rebound hyperuricaemia.

## Introduction

Febuxostat is a highly selective and potent inhibitor of xanthine oxidase that has been developed to treat patients who have an insufficient response or intolerance to allopurinol.^1 2^ However, the adverse cardiovascular (CV) effects of febuxostat have been a concern throughout its development, and the Cardiovascular Safety of Febuxostat or Allopurinol in Patients with Gout (CARES) trial was performed as a requirement by the US Food and Drug Administration (FDA) for patients with gout and major CV disease.^3^ The CARES trial suggested that the incidence of CV death was higher in the febuxostat group than in the allopurinol group.^3^ Consequently, the FDA added a boxed warning to febuxostat regarding the increased mortality risk. However, the urate-lowering efficacy and safety of febuxostat in the treatment of the hyperuricemia of gout (CONFIRMS) trial and the Long-term cardiovascular safety of febuxostat compared with allopurinol in patients with gout (FAST) trial demonstrated that febuxostat was not associated with an increased risk of death or serious adverse CV events compared with allopurinol.^4 5^ In addition, the risk of CV events and mortality between patients treated with febuxostat and allopurinol were similar in the subsequent nationwide retrospective cohort studies.^6 7^ Thus, the higher risk of CV events and mortality from febuxostat than from allopurinol remains controversial.^8^

Regarding the increased risk of CV mortality associated with febuxostat in the CARES trial, there are previous studies that reanalysed the CARES trial data to calculate the mortality rates on the basis of the median duration of exposure to study drugs (table S12 of the previous work^3^).^9^ In our reanalysis, a 40-fold increase in mortality was observed after allopurinol or febuxostat was discontinued. The sharp increase in mortality was thought to be associated with rapid changes in the serum uric acid levels because of the drug discontinuation (rebound hyperuricaemia), which is a known risk factor for acute gout attacks. Accordingly, we hypothesised that some CV events may share the same mechanism as those of acute gout attacks (acute inflammation induced by monosodium urate (MSU) crystals in the CV system).^9^

We assumed that if fluctuations in uric acid levels triggered CV events, the increase would be rapid in the initial stage after drug discontinuation. Thus, we investigated the incidence of CV events and death in the initial stage after discontinuation of febuxostat or allopurinol using the CARES trial data. Moreover, the most important criticism of our previous interpretation is that allopurinol or febuxostat may have been discontinued owing to the worsening of the systemic condition, resulting in increased deaths after discontinuing allopurinol or febuxostat (the ‘sick-stopper’ effect^10 11^). Therefore, we reanalysed the data from patients with no abnormal clinical or vital signs during any of the study visits in the CARES trial to ascertain the temporal relationship of CV events/death.

## Methods

### Study design and population

The CARES trial was a multicentre, randomised, double-blind non-inferiority trial. The study population comprised 6198 patients with gout and a history of major CV disease before randomisation. Similar to the CARES trial, we used the data of 6190 patients in a modified intention-to-treat analysis. The included patients were examined for concurrent conditions, vital signs/weight at scheduled study visits, the end of the study and early termination; the patients also underwent physical examination and 12-lead electrocardiography (protocol of the CARES trial^3^). Subjects who have withdrawn from study medication treatment but have not withdrawn consent will be followed until the subject experiences a CV event that is positively adjudicated as a MACE or until the study concludes. Subjects will be contacted by telephone every 2 months (±10 days) to determine if any potential CV events have occurred. We had access to data of the CARES trial through the company Vivli, and we reanalysed the CARES trial data with no financial support from any company.

### Outcomes

As in the CARES trial, we investigated MACE, such as CV death, non-fatal myocardial infarction, non-fatal stroke or urgent revascularisation for unstable angina, as primary endpoints. MACE and cause of death were adjudicated by an independent central endpoints committee, the members of which were unaware of the treatment assignments according to the same protocol as in the CARES trial. Using the CARES trial data, we compared the MACE that occurred during study drug administration (including day 1 after the last medication) and after study drug discontinuation (from day 2 after the last medication), including or excluding patients with gout with abnormal clinical or vital signs (physical examination findings, ECG findings, body temperature, systolic and diastolic blood pressure, and pulse/beats per minute) at the study visits to minimise ‘sick-stopper’ effects, that sicker lifestyles often accompany non-adherent behaviours.^10^ Patients who experienced a MACE during study drug administration were excluded from the study drug discontinuation group. In addition, we compared the incidence of adverse events leading to CV death and non-CV death after drug discontinuation, excluding patients with gout with abnormal clinical or vital signs at the study visits in order to minimise ‘sick-stopper’ effects.^10–12^ We investigated up to 1, 3 and 6 months of discontinuation of the study drug to best consider the period during which uric acid is rapidly increased and its effects are most likely to occur.

### Statistical analysis

Data are presented as mean±SD, median (IQR) for continuous variables or as the number (%) of subjects for categorical variables. Comparisons of baseline characteristics among the three groups were made using the Pearson’s χ^2^ test, Fisher’s exact test, analysis of variance or Kruskal-Wallis test according to the type of variable (table 1).

**Table 1:** Baseline characteristics of all study patients

Characteristics	Modified ITT (n=6190)				
	No CV event* (n=5534)	CV event*		P value (no CV event group vs CV event group)*	P value (during vs after)†
		During administration† (n=448)	After discontinuation† (n=208)		
Treatment, n (%)				0.5365	0.9958
Febuxostat	2763 (49.9)	223 (49.8)	112 (53.8)		
Allopurinol	2771 (50.1)	225 (50.2)	96 (46.2)		
Median age, years (IQR)	64.0 (58.0–70.0)	66.0 (60.0–72.0)	69.0 (62.0–75.0)	<0.0001	0.0054
Age≥65, n (%)	2704 (48.9)	252 (56.4)	140 (67.3)	<0.0001	0.0237
Male, n (%)	4627 (83.6)	396 (88.4)	173 (83.2)	0.0283	0.1998
Body mass index	33.5±6.9	33.4±6.4	34.3±7.9	0.2502	0.2894
Median duration of gout, years (IQR)	7.8 (3.1–17.2)	9.9 (3.4–20.8)	5.4 (2.3–15.5)	0.0025	0.0030
Median number of gout flares (IQR)	2.0 (1.0–4.0)	3.0 (1.0–5.0)	2.0 (1.0–4.0)	0.0013	0.1545
Median number of tophi (IQR)	2.0 (1.0–4.0)	2.0 (1.0–3.0)	2.0 (2.0–5.0)	0.0586	0.0434
Baseline serum urate level, mean±SD	8.7±1.7	9.0±1.7	9.4±1.8	<0.0001	0.0104
Cardiovascular risk factors and history, n (%)					
DM with small-vessel disease	2175 (39.3)	144 (32.1)	87 (41.8)	0.0077	0.0470
Hypertension	5091 (92.0)	426 (95.1)	198 (95.2)	0.0175	1.0000
Hyperlipidaemia	4789 (86.5)	407 (90.8)	184 (88.5)	0.0270	1.0000
Myocardial infarction	2072 (37.4)	251 (56.0)	105 (50.5)	<0.0001	0.5537
Hospitalisation for unstable angina	1473 (26.6)	177 (39.5)	74 (35.6)	<0.0001	1.0000
Coronary revascularisation	1955 (35.3)	245 (54.7)	111 (53.4)	<0.0001	1.0000
Cerebral revascularisation	102 (1.8)	13 (2.9)	8 (3.8)	0.0450	1.0000
Congestive heart failure	1024 (18.5)	152 (33.9)	77 (37.0)	<0.0001	1.0000
Stroke	740 (13.4)	96 (21.4)	34 (16.3)	<0.0001	0.3858
Peripheral vascular disease	666 (12.0)	73 (16.3)	48 (23.1)	<0.0001	0.1114

P value by χ^2^ test, Fisher’s exact test, analysis of variance or Kruskal-Wallis test.

*, † Adjusted p value by χ^2^ test, Fisher’s exact test with Bonferroni adjustment method, Dwass, Steel, Critchlow–Fligner multiple comparison or Tukey’s multiple comparison method.

DM, diabetes mellitus; ITT, intention-to-treat; sUA, serum uric acid.

The main analyses relied on time‐to‐event methods and self-controlled case series analysis. The cohort entry (time 0) corresponded to the date of randomisation, and individuals were followed until the date of the occurrence of MACE, death or last contact. The study exposure was the discontinuation of the study drug and was considered as a time-varying variable (subjects who discontinued the study drug contributed to the unexposed person-time before discontinuation of the study drug). MACEs that occurred up until day 1 after the last medication were included in the ‘during administration’ status. For subjects who had no MACE during the drug administration period, the day 1 after the last medication was changed to time-zero, and follow-up ended when a MACE occurred. In this case, the curves generated by the extended Kaplan-Meier method do not represent fixed cohorts of subjects, as subjects can contribute to different curves at different time points during follow-up.

The person-years for each subject according to exposure status (during administration or after discontinuation of the study drug) were calculated from the index date to their end of follow-up. The crude incidence rates (IRs) of MACE were calculated as the number of MACEs per 100 person-years. The IR ratio (IRR) for MACE was calculated as the IR of MACE in the discontinuation of the study drug period compared with the IR of MACE during the administration period. The 95% CIs of the IRs and IRR were derived from Poisson regression models. A Kaplan-Meier survival curve of the probability of developing a MACE was constructed according to exposure status (during administration or after discontinuation of the study drug). Time-varying Cox models were used to evaluate the association between ‘discontinuation of study drug’ status and the incidence of MACE. HR are presented with corresponding 95% CIs.^13^ To investigate the effect of discontinuation of the study drug, the risk for MACE was compared according to increases in the difference between last measured and initial uric acid levels for discontinuation periods using Cox regression analysis for gout patients who took febuxostat or allopurinol for more than 1 year.

For the death outcome, cohort entry (time 0) corresponded to the date of the last medication. Individuals were followed up until the date of death or the last contact date. All-cause mortality was calculated using the Kaplan-Meier method. Cumulative incidence functions were used to estimate the CV mortality to account for the competing risk of non-CV death.

All analyses were performed using SAS V.9.4 (SAS Institute). All statistical tests conducted were two-sided, and p values <0.05 were considered statistically significant.

## Results

### Baseline characteristics of the subjects

Among 6190 patients in a modified intention-to-treat analysis, MACE occurred in 448 patients (7.2%) during the administration of the study drugs and in 208 patients (3.3%) after discontinuation of the study drugs. Patients who had their first MACE after discontinuation of the study drugs showed significantly higher baseline serum urate levels and the number of tophi, a significantly shorter duration of gout than did patients with their first MACE during study drug administration (table 1). Online supplemental table 1 depicts cardiovascular events during drug administration and after discontinuation according to the primary reasons behind the study drug not being completed. Among 2315 patients with no abnormal clinical or vital signs during the study visits, MACE occurred in 138 patients (5.9%) during study drug administration compared with 64 patients (2.7%) after study drug discontinuation. The baseline characteristics of the patients are presented in online supplemental table 2.

10.1136/rmdopen-2021-001944.supp1 Supplementary data

### Risk of MACEs between during administration and after discontinuation

**Figure 1:**
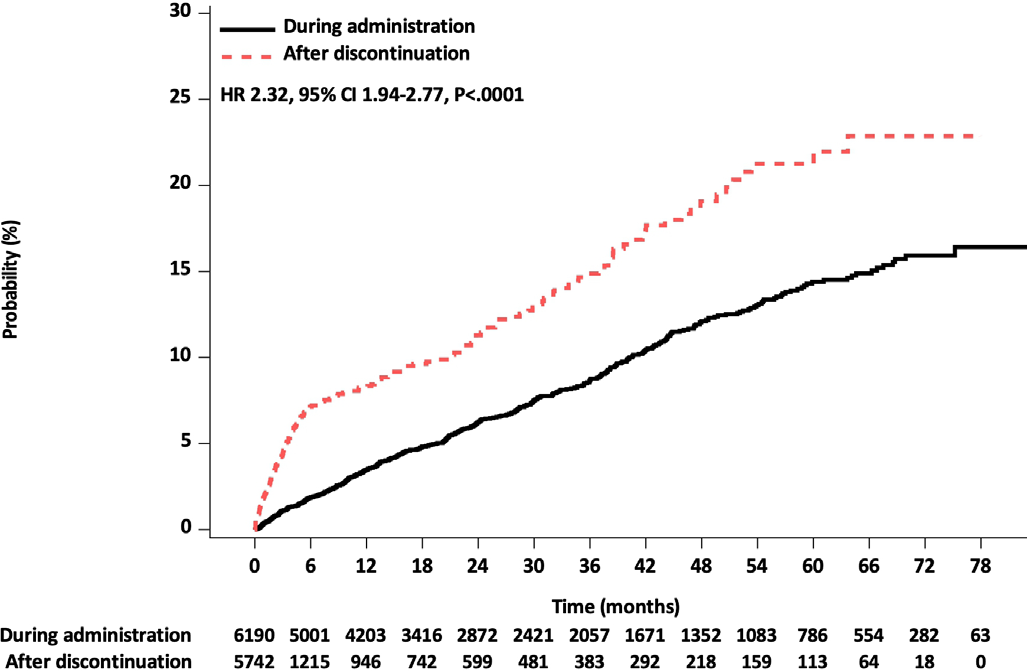
Cumulative Kaplan-Meier estimates of the time to the first occurrence of major adverse cardiovascular events (all study patients).

Among 6190 patients in a modified intention-to-treat analysis, the incidence rate per 100 person-years for MACE was 3.11 during administration and 6.71 after discontinuation of the study drugs. A higher risk of MACE was observed after discontinuation of the study drugs than that observed during administration (HR 2.32; 95% CI 1.94 to 2.77; p<0.0001) (figure 1 and table 2). For 12 months, a similar risk of MACE was observed between febuxostat and allopurinol after discontinuation (online supplemental figure 1). In addition, as a sensitivity analysis, we compared the incidence of MACE in the initial stage between after febuxostat or allopurinol initiation and after discontinuation in various situations (online supplemental figure 2 and table 3). In these 6-month analyses, a consistently increased risk of MACE was observed after discontinuation compared with that during administration. In the analysis of patients with no abnormal clinical or vital signs during the study visits to minimise ‘sick-stopper’ effects, even if patients who were excluded because of the presence of abnormal clinical or vital signs had MACE that occurred during administration are only included in the ‘during administration’ group, the incidence of MACE was significantly higher after discontinuation of the study drugs than that during administration of the study drugs within 1 month (HR 4.90; 95% CI 3.08 to 7.80), 3 months (HR 3.42; 95% CI 2.37 to 4.91) and 6 months (HR 2.92; 95% CI 2.16 to 3.96) (online supplemental figure 2D and table 3D). Moreover, as the follow-up duration after discontinuation for the completed group was shorter compared with that of the drug discontinuation group, survival curves for MACE between during drug administration and after discontinuation under the assumption that participants who completed the trial did not have any MACEs were determined, as shown in online supplemental table 1. The incidence of MACE was significantly higher after discontinuation of the study drugs than that during the administration of the study drugs within 1 month (HR 4.03; 95% CI 2.94 to 5.52), 3 months (HR 3.01; 95% CI 2.38 to 3.81) and 6 months (HR 2.44; 95% CI 1.99 to 2.99) (online supplemental figure 3).

**Table 2:** Comparative risk of MACEs between during administration and after discontinuation of the study drug

Last study drug stop	Events (n)	Person-years	Incidence rates per 100 person-years	IRR (95% CI)	P value	HR (95% CI)	P value
During administration	448	14 424	3.11 (2.83–3.41)	1 (Ref)		1 (Ref)	
After discontinuation	208	3101	6.71 (5.86–7.68)	2.16 (1.83 to 2.55)	<0.0001	2.32 (1.94 to 2.77)	<0.0001

IRR (incidence rate ratio) by Poisson regression.

HR (hazard ratio) by Cox regression with time-varying covariate.

MACEs, major adverse cardiovascular events.

### Changes in serum uric acid level and MACE after discontinuation of the study drugs

A portion of patients were tested for uric acid levels after drug discontinuation. Uric acid levels rebounded after 2–3 weeks after drug discontinuation (online supplemental figure 4). The number of patients with at least 1 year of drug administration was 4195, among which, 359 patients developed MACE during the administration of the study drugs. After excluding the 359 patients with MACE during drug administration, the number of patients who did not develop MACE during drug administration was 3836, among which, 97 patients had MACE after drug discontinuation (online supplemental table 4). Factors associated with higher MACE risk after drug discontinuation included age, body mass index and changes in serum uric acid levels (per 1 mg/dL increase) from baseline to last measurement before discontinuation (HR 1.14; 95% CI 1.04 to 1.26, table 3). On the other hand, there was no association between MACEs during drug administration and changes in serum uric acid levels from baseline to last measurement before discontinuation (HR 0.99; 95% CI 0.95 to 1.04, online supplemental table 5).

**Table 3:** Multivariable Cox regression analysis of MACE in patients with gout with more than 1 year of febuxostat or allopurinol administration after drug discontinuation

	Unadjusted		Adjusted	
	HR (95% CI)	P value	HR (95% CI)	P value
Treatment				
Allopurinol	1 (Ref)		1 (Ref)	
Febuxostat	1.20 (0.81 to 1.80)	0.3637	1.11 (0.73 to 1.67)	0.6343
Age (per 10 unit increase)	1.63 (1.28 to 2.06)	<0.0001	1.48 (1.08 to 2.02)	0.0143
Male	0.92 (0.54 to 1.57)	0.7573	0.98 (0.55 to 1.74)	0.9406
Body mass index	1.02 (1.00 to 1.05)	0.0637	1.03 (1.00 to 1.06)	0.0448
Race or ethnic group				
White	1 (Ref)		1 (Ref)	
Black or African-American	0.64 (0.34 to 1.20)	0.1626	0.69 (0.34 to 1.40)	0.3046
Others	0.42 (0.17 to 1.04)	0.0604	0.58 (0.22 to 1.53)	0.2682
Smoker				
Never smoked	1 (Ref)		1 (Ref)	
Current smoker	1.16 (0.60 to 2.24)	0.6549	1.54 (0.77 to 3.10)	0.2258
Ex-smoker	1.18 (0.75 to 1.86)	0.4712	0.96 (0.57 to 1.62)	0.8920
Drink				
Never drank	1 (Ref)		1 (Ref)	
Current drinker	0.82 (0.50 to 1.36)	0.4469	0.90 (0.51 to 1.58)	0.7198
Ex-drinker	0.93 (0.54 to 1.62)	0.7975	1.02 (0.54 to 1.90)	0.9572
Baseline glucose (per 10 unit increase)	1.02 (0.98 to 1.06)	0.3317	1.03 (0.98 to 1.08)	0.3041
Baseline LDL (per 10 unit increase)	1.00 (0.95 to 1.06)	0.9926	1.04 (0.98 to 1.10)	0.2314
Baseline SBP (per 10 unit increase)	1.10 (0.98 to 1.22)	0.0997	1.12 (0.98 to 1.28)	0.1018
Baseline DBP (per 10 unit increase)	0.93 (0.77 to 1.12)	0.4319	0.96 (0.76 to 1.20)	0.7049
Change in sUA levels* (per 1 mg/dL increase)	1.20 (1.09 to 1.31)	0.0001	1.14 (1.04 to 1.26)	0.0069
Stage of chronic kidney disease (eGFR)				
Stage 1 (90+)	1 (Ref)		1 (Ref)	
Stage 2 a (75–89)	1.86 (0.60 to 5.78)	0.2805	1.56 (0.49 to 4.97)	0.4486
Stage 2b (60–74)	1.65 (0.55 to 4.90)	0.3692	1.20 (0.39 to 3.71)	0.7453
Stage 3 a (45–59)	2.81 (0.99 to 8.00)	0.0525	1.58 (0.52 to 4.76)	0.4192
Stage 3b (30–44)	5.44 (1.92 to 15.40)	0.0014	2.58 (0.83 to 8.03)	0.1008
Stage 4 (15–29)	6.36 (1.59 to 25.44)	0.0089	3.41 (0.78 to 14.96)	0.1034
Baseline uroprotein				
Negative+Trace	1 (Ref)		1 (Ref)	
+1	0.94 (0.45 to 1.94)	0.8593	0.95 (0.44 to 2.05)	0.9037
+2–+4	1.34 (0.70 to 2.59)	0.3805	1.03 (0.49 to 2.18)	0.9431
Duration of gout (per 10 unit increase)	1.02 (0.87 to 1.21)	0.7757	0.99 (0.83 to 1.17)	0.8934
Presence of tophi	1.17 (0.74 to 1.84)	0.5023	1.23 (0.76 to 2.01)	0.4023
Cardiovascular risk factors and history				
DM with small-vessel disease	1.00 (0.66 to 1.50)	0.9819	0.80 (0.49 to 1.31)	0.3733
Hypertension	3.93 (0.97 to 15.94)	0.0555	2.84 (0.68 to 11.84)	0.1508
Hyperlipidaemia	1.13 (0.63 to 2.03)	0.6781	0.74 (0.40 to 1.39)	0.3530
Myocardial infarction	1.36 (0.91 to 2.04)	0.1301	1.14 (0.71 to 1.81)	0.5876
Hospitalisation for unstable angina	1.00 (0.65 to 1.55)	0.9881	0.85 (0.53 to 1.37)	0.5047
Coronary revascularisation	1.64 (1.10 to 2.44)	0.0151	1.39 (0.87 to 2.23)	0.1650
Cerebral revascularisation	3.20 (1.17 to 8.73)	0.0230	2.34 (0.81 to 6.77)	0.1158
Congestive heart failure	1.99 (1.30 to 3.04)	0.0015	1.44 (0.89 to 2.31)	0.1372
Stroke	0.99 (0.56 to 1.75)	0.9752	0.91 (0.50 to 1.67)	0.7710
Peripheral vascular disease	1.69 (1.03 to 2.76)	0.0372	1.51 (0.89 to 2.55)	0.1255
Initial prophylactic medication				
Colchicine 0.6 mg once a day	1 (Ref)		1 (Ref)	
Naproxen 250 mg two times per day+PPI	0.49 (0.21 to 1.11)	0.0871	0.73 (0.31 to 1.71)	0.4682
Other+none	1.18 (0.48 to 2.92)	0.7145	1.40 (0.54 to 3.63)	0.4873

*Change in sUA levels: determined by calculating the difference between baseline and last measured serum uric acid prior to drug discontinuation

DBP, diastolic blood pressure; DM, diabetes mellitus; LDL, low-density lipoprotein; PPI, proton-pump inhibitors; SBP, systolic blood pressure; sUA, serum uric acid.

### Mortality between cardiovascular versus non-cardiovascular death

**Figure 2:**
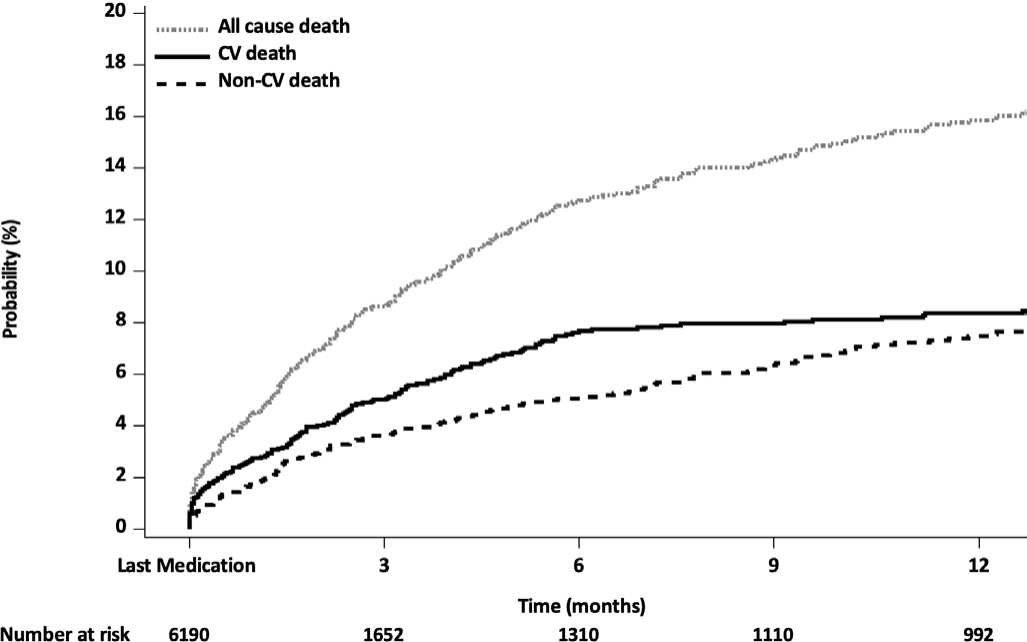
Cumulative Kaplan-Meier estimates of the time to death after the last medication (including deaths induced by adverse events before the last medication).

Among the total 6190 patients, 442 patients died. The CV mortality rate was significantly higher than the non-CV mortality rate within 1 month after discontinuation (44.0% (n=103/234) vs 29.3% (n=61/208), p=0.0014), 3 months after discontinuation (63.7% (n=149/234) vs 47.1% (n=98/208), p=0.0005) and 6 months after discontinuation (82.1% (n=192/234) vs 58.7% (n=122/208), p<0.0001) (figure 2, online supplemental figure 5 and online supplemental table 6). Table 4, online supplemental table 7, 8 and figure 6 depict CV death and mortality in patients with or without no abnormal clinical or vitals signs during the study visits, excluding death induced by adverse events that occurred up to 1 day after last medication.

**Table 4:** Comparative mortality between cardiovascular versus non-cardiovascular death (excluding death adjudicated by adverse events that occurred up to 1 day after the last medication)

	Total (n=442)	CV death (n=234)	Non-CV death (n=208)	P value
Death within 1 month, n (%)				0.1091
No	402 (91.0)	208 (88.9)	194 (93.3)	
Yes	40 (9.0)	26 (11.1)	14 (6.7)	
Death within 3 months, n (%)				0.0151
No	336 (76.0)	167 (71.4)	169 (81.3)	
Yes	106 (24.0)	67 (28.6)	39 (18.8)	
Death within 6 months, n (%)				0.0001
No	277 (62.7)	127 (54.3)	150 (72.1)	
Yes	165 (37.3)	107 (45.7)	58 (27.9)	

P value by χ^2^ test.

CV, cardiovascular.

## Discussion

In this reanalysis of the CARES trial data of patients with gout and established CV disease, a sudden increase in MACE, CV death compared with non-CV death, and adverse events adjudicated as CV death compared with non-CV death were observed in the initial stage after discontinuation of the study drugs. In addition, changes in the serum uric acid levels from baseline to the last measurement before discontinuation were significantly associated with higher MACE risk after drug discontinuation.

### MACE after discontinuation of the study drugs in FAST trials

Similar to our results, a threefold increase in CV events after study drug discontinuation was observed in the long-term cardiovascular safety of febuxostat compared with allopurinol in patients with gout (the FAST trial), which was calculated on the basis of the median duration of exposure to study drugs (0.31 CV event/day; 413 CV events/1324 days (median duration of on-treatment follow-up) versus 0.90 CV event/day; 128 CV events/143 days (median duration all follow-up–median duration of on-treatment follow-up)).^5^ The FAST trial was able to follow-up study patients until the end of the trial by telephone and other personal contact and by record linkage to national hospitalisation and death records (except in the small proportion of patients who withdrew completely). In addition, there were lower rates of treatment discontinuation (32.4% in the febuxostat group and 16.5% in the allopurinol group) and much better rates of patient follow-up, with only 5.8% of patients withdrawing from all follow-up in the FAST trial.^5^ Considering these two points, an untold message from the FAST study may be increases in cardiovascular events after drug discontinuation. In the CARES and FAST trials, the cardiovascular risk of febuxostat administration compared with allopurinol administration showed different results. However, an increase in cardiovascular events was observed after study drug discontinuation in both studies.

### MSU crystals in the vasculature and CV gout attacks

Recent studies with dual-energy CT (DECT) have demonstrated the deposition of MSU crystals in the vasculature of 86%–88% of patients with gout,^14 15^ while MSU crystals have also been observed in the coronary arteries, aortic plaques and various tissues.^15 16^

Since inflammation in the vasculature is known to trigger plaque rupture,^17 18^ it is likely that inflammation in the blood vessels can cause CV events. A sudden change in serum uric acid levels as a result of the withdrawal or initiation of allopurinol or febuxostat, or other risk factors for acute gout attacks, could induce the formation of MSU crystals in the blood vessels.^19^ It is thought that MSU crystals might cause acute inflammation in the blood vessels as well as intermittent gout attacks in the joints, resulting in plaque rupture and CV events (CV gout attacks). Therefore, a situation in which a gout attack is triggered might also trigger CV gout attacks; this could explain the increase in CV events and mortality after discontinuation of allopurinol and febuxostat in this reanalysis of the CARES trial, as well as the sudden increase in CV events before and after the initiation of allopurinol and febuxostat in a nationwide cohort from our other study.^20^ Furthermore, higher MACE risk after drug discontinuation was observed on increasing levels of the difference between baseline and last measured uric acid levels prior to drug discontinuation. This difference may act as a surrogate marker for the degree of rebound hyperuricaemia.^21^ Considering the fact that uric acid levels increase within 1–2 weeks after drug discontinuation,^21^ rebound hyperuricaemia that occurs after drug discontinuation might be considered a significant risk factor for MACE. This significant association of difference in baseline and last measured uric acid levels prior to drug discontinuation and MACE risk after drug discontinuation is an important result in supporting our hypothesis.

Recently, Pascart *et al* suggested that DECT-based vascular MSU-coded plaques located in arteries may be artefacts rather than MSU crystals.^22^ The tophi observed in the DECT data may not all be tophi and may contain some false positives. In a previous study,^15^ MSU crystals were observed in the biopsy of tissues in which tophi signals were observed from the DECT data. Moreover, considering the results of a recent study in which patients with gout had a greater number and volume of tophi in the CV system on DECT compared with controls,^23^ tophi signals from DECT are likely associated with aggregated MSU crystals formed in the CV system. Therefore, tophi in CV systems could contribute to the development of CV diseases.

According to the protocol of the study, colchicine was administered for 6 months, after which the medication was discontinued. Therefore, MACE development was likely affected within 6 months after drug initiation. However, the effect of colchicine on reducing MACE risk was 23%~31%,^24 25^ which is smaller than the increased MACE risk observed in our study. Therefore, while colchicine may have affected MACE risk, it is thought to be not substantial. Furthermore, while methotrexate, which can affect inflammation, did not affect MACE risk,^26^ the fact that colchicine was associated with MACE risk suggests that MSU crystals might affect MACE risk. Colchicine reduces inflammation associated with MSU crystals, and the *NEJM* study participants may be high risk CV patients with the possibility of having non-symptomatic hyperuricaemia and gout. Therefore, there may be the possibility that colchicine reduced MACE risk in part via reducing inflammation from MSU crystals.

Considering these points, MSU crystal deposition, uric acid fluctuations and low compliance of patients to hypouricaemic agents may have a greater impact than has been previously understood. Further, hyperuricaemia and MSU crystals, like dyslipidaemia, might play important roles in the mechanism of CV pathogenesis. Gout may not be limited to joint diseases and requires a paradigm shift where it can be regarded as a systemic disease.^27^

### Limitations

This study has limitations in addition to those inherent to post hoc analysis of the CARES trial. It is known that the CARES trial is composed of a relatively large portion of patients who did not complete the trial. Therefore, there is the possibility that patients did not complete the trial due to the worsening of systemic conditions. Therefore, we believe that one of the major limitations of this study is the inability to exclude the possibility of the ‘sick stopper effect’. To minimise the impact of the ‘sick-stopper’ effect, we investigated CV mortality and adverse events adjudicated as between CV death in patients with or without abnormal clinical or vital signs during all study visits, using data at the point of systemic condition worsening. However, treatment information on patients who have dropped out or discontinued treatment is lacking. Despite this, our study showed consistent increases in CV events 6 months after drug discontinuation. We believe that this 6-month period is too long to be solely attributed to the sick stopper effect.

In addition, changes in serum uric acid levels from baseline to the last measurement before discontinuation was significantly associated with higher MACE risk after drug discontinuation. On the other hand, there was no association between MACEs during drug administration and changes in serum uric acid levels from baseline to the last measurement before discontinuation. Furthermore, a sensitivity analysis on the primary safety endpoint during 1 month after treatment discontinuation was performed.

Another important limitation of the current study is the inability to obtain all clinical information for all patients after drug discontinuation. However, we were able to analyse continuous follow-up data that were gathered every 2 months after drug discontinuation because this study was based on the data from an randomized controlled trial. Therefore, MACEs could be determined in a relatively large number of patients with drug discontinuation, as shown in online supplemental table 1. In contrast, information on MACEs among participants who completed the trial was lacking. Therefore, we conducted the analysis under the assumption that those who completed the trial did not have any MACEs. Despite this, MACEs increased within the first 6 months after drug discontinuation, as shown in online supplemental figure 3. Moreover, we analysed MACEs among the drug discontinuation patients in various sensitivity and stratified analyses.

Therefore, we believe there is some evidence on the increased MACE risk on drug discontinuation. This may be particularly important as ethical considerations make it difficult to conduct prospective studies on determining MACE risk on drug discontinuation.

## Conclusions

Previously, the CARES trial suggested that the incidence of all-cause mortality and CV death was higher in the febuxostat group than in the allopurinol group. However, the real message from the CARES trial was that increases in the incidence of MACE, CV death and adverse events leading to CV death were observed after discontinuing febuxostat or allopurinol than during administration.

## Data Availability

Data may be obtained from a third party and are not publicly available.
