# Supplementary material for: Increased risk of cardiovascular events and death in the initial phase after discontinuation of febuxostat or allopurinol: another story of the CARES trial
Source: RMD Open. 2022 Jun 22;8(2):e001944. doi: 10.1136/rmdopen-2021-001944 (PMC9226988; doi:10.1136/rmdopen-2021-001944)
Supplement: Supplementary data [file rmdopen-2021-001944supp001.pdf]

Supplementary Table 1. Cardiovascular events during drug administration and after discontinuation according to primary reasons behind study drug not being completed.

Characteristics	Modified ITT (n = 6190)		
		CV event*	
	No CV event* (n = 5534)	During administration† (n = 448)	After discontinuation† (n = 208)
Primary Reason Study Drug Not Completed			
Completed study drug	2529 (45.7)	158 (35.3)	2 (1.0)
Discontinued study drug			
- Pretreatment Event/Adverse Event	615 (11.1)	161 (35.9)	122 (58.7)
- Major Protocol Deviation	186 (3.4)	5 (1.1)	9 (4.3)
- Lost to Follow-Up	397 (7.2)	17 (3.8)	4 (1.9)
- Voluntary Withdrawal	1267 (22.9)	64 (14.3)	34 (16.3)
- Other	540 (9.8)	43 (9.6)	37 (17.8)
Last medication date ~ last contact date (days), Median (IQR)			
Completed study drug	1 (1 - 1)	1 (1 - 1)	63 (35 - 91)
Discontinued study drug			
- Pretreatment Event/Adverse Event	158 (16 - 757)	6 (1 - 86)	113 (46 - 444)
- Major Protocol Deviation	111 (1 - 779)	78 (21 - 249)	1157 (851 - 1395)
- Lost to Follow-Up	1 (0 - 57)	5 (1 - 234)	428 (311 - 862)
- Voluntary Withdrawal	61 (1 - 377)	112 (10 - 196)	545 (189 - 1091)
- Other	40 (1 - 558)	3 (1 - 130)	149 (73 - 707)

Supplementary Table 2. Baseline characteristics of patients with no abnormal clinical or vital signs during the study visits

Characteristics	No CV event* (n = 2315)	CV event*		P-value*	P-value†
		During administration† (n = 138)	After discontinuation† (n = 64)		
Treatment, n (%)				0.901	1.0000
Febuxostat	1164 (50.3)	70 (50.7)	34 (53.1)		
Allopurinol	1151 (49.7)	68 (49.3)	30 (46.9)		
Median age, year (IQR)	63.0 (58.0–70.0)	65.0 (59.0–71.0)	68.0 (62.0–73.0)	0.0003	0.0809
Age ≥ 65, n (%)	1060 (45.8)	71 (51.8)	43 (67.2)	0.0015	0.1217
Male, n (%)	1954 (84.4)	125 (90.6)	57 (89.1)	0.0923	1.0000
Median duration of gout, year (QI)	7.7 (3.2–17.2)	8.8 (3.3–18.7)	6.8 (2.3–19.7)	0.6880	0.8179
No. of sUA tests for each subject collected from the end of the first year of treatment until 1 day after last dose date	5.0 (3.0–8.0)	6.0 (3.0–9.0)	4.0 (3.0–6.0)	0.0136	0.0272
Average sUA value for each subject collected from the end of the first year of treatment until 1 day after last dose date	5.3 (4.6–6.2)	5.4 (4.7–6.0)	5.3 (4.6–6.4)	0.8385	0.9125
Baseline serum urate level, mean ± SD	8.6 ± 1.6	8.9 ± 1.6	9.2 ± 1.7	0.0017	0.4299
Median no. of gout flares (IQR)	2.0 (1.0–4.0)	3.0 (2.0–5.0)	2.0 (2.0–5.0)	0.0257	0.8516
Presence of tophi, n (%)	468 (20.2)	23 (16.7)	18 (28.1)	0.1687	0.1788
Median no. of tophi (IQR)	2.0 (1.0–4.0)	2.0 (1.0–3.0)	2.0 (2.0–5.0)	0.2528	0.2473
Median body weight, kg (IQR)	96.8 (84.0–112.6)	100.9 (91.0–121.0)	104.1 (91.6–119.3)	0.0007	0.6873
Body mass index	33.4 ± 6.8	34.0 ± 6.7	35.6 ± 8.7	0.0225	0.2639
Race or ethnic group, n (%)				<0.0001	0.1728
American Indian or Alaska Native	253 (10.9)	0 (0.0)	4 (6.3)		
Asian	75 (3.2)	3 (2.2)	2 (3.1)		
Black or African American	424 (18.3)	17 (12.3)	9 (14.1)		
Native Hawaiian or Other Pacific Islander	11 (0.5)	2 (1.4)	0 (0.0)		
White	1542 (66.6)	114 (82.6)	49 (76.6)		
Other	10 (0.4)	2 (1.4)	0 (0.0)		
Cardiovascular risk factors and history, n (%)					
DM with small-vessel disease	927 (40.0)	45 (32.6)	30 (46.9)	0.1123	0.1526
Hypertension	2084 (90.0)	129 (93.5)	62 (96.9)	0.0832	1.0000

Hyperlipidemia	1985 (85.7)	128 (92.8)	56 (87.5)	0.0650	0.6682
Myocardiac infaction	815 (35.2)	73 (52.9)	27 (42.2)	<0.0001	0.4698
Hospitalization for unstable angina	580 (25.1)	55 (39.9)	18 (28.1)	0.0005	0.3192
Coronary revascularization	783 (33.8)	74 (53.6)	29 (45.3)	<0.0001	0.8149
Cerebral revascularization	30 (1.3)	3 (2.2)	4 (6.3)	0.0141	0.6332
Congestive heart failure	365 (15.8)	37 (26.8)	19 (29.7)	<0.0001	1.0000
Stroke	288 (12.4)	30 (21.7)	9 (14.1)	0.0066	0.5953
Peripheral vascular disease	264 (11.4)	18 (13.0)	12 (18.8)	0.1720	0.8659
Median estimated creatinine clearance					
Stage 1 or 2 chronic kidney disease	74.0 (66.0–84.0)	72.0 (66.0–82.0)	71.0 (66.0–82.0)	0.5480	0.9822
Stage 3 chronic kidney disease	46.0 (40.0–53.0)	46.0 (38.0–53.0)	43.0 (37.0–52.0)	0.2617	0.5824
Stage of chronic kidney disease, no./total no. (%)				<0.0001	0.1112
Stage 1 or 2	1221/2311 (52.8)	60/138 (43.5)	18/64 (28.1)		
Stage 3	1090/2311 (47.2)	78/138 (56.5)	46/64 (71.9)		
Primary reason study drug not completed				<0.0001	<0.0001
Completed study drug	1297 (56.0)	53 (38.4)	0 (0.0)		
Discontinued study drug					
Adverse events	138 (6.0)	36 (26.1)	36 (56.3)		
Major protocol deviation	77 (3.3)	1 (0.7)	2 (3.1)		
Lost to follow-up	109 (4.7)	6 (4.3)	1 (1.6)		
Voluntary withdrawal	470 (20.3)	27 (19.6)	9 (14.1)		
Other	224 (9.7)	15 (10.9)	16 (25.0)		
Median duration of treatment, day (IQR)	833.0 (457.0–1454.0)	1238.5 (676.0–1752.0)	729.5 (394.0–1109.0)	<0.0001	<0.0001
Last clinical sign date – last medication date (days), median (IQR)	-1.0 (-1.0–0.0)	0.0 (-1.0–182.0)	16.0 (0.0–178.0)	<0.0001	0.1090
Last vital date – last medication date (days), median (IQR)	-1.0 (-1.0–0.0)	0.0 (-1.0–103.0)	0.0 (0.0–50.5)	<0.0001	0.5928
Last medication date – last contact date (days), median (IQR)	1.0 (1.0–24.0)	1.0 (1.0–68.0)	112.5 (35.5–407.0)	<0.0001	<0.0001
Last clinical sign date – last medication date (days), median (IQR)					
Completed study drug	-1 (-1–[-1])	-1 (-1–[-1])	-	0.4708	
Discontinued study drug					
Adverse events	28 (0–182)	162 (46–236)	101 (0–185)	0.0107	0.1156
Major protocol	-1 (-1– 0)	290 (290–290)	4 (0–8)	0.0901	0.4385

deviation					
Lost to follow-up	181 (4–199)	150 (90–198)	379 (379–379)	0.2467	0.2909
Voluntary withdrawal	0 (-1–196)	88 (0–200)	0 (0–60)	0.2991	0.1798
Other	0 (-1–171)	74 (-1–296)	0 (0–105)	0.4384	0.9507
Last vital date – last medication date (days), median (IQR)					
Completed study drug	-1 (-1–[-1])	-1 (-1–[-1])	-	0.5013	
Discontinued study drug					
Adverse events	14 (0–74)	97 (30–158)	0 (0–46)	0.0001	0.0005
Major protocol deviation	-1 (-1–0)	104 (104–104)	4 (0–8)	0.0751	0.4385
Lost to follow-up	0 (0–151)	110 (0–171)	199 (199–199)	0.2863	0.4172
Voluntary withdrawal	0 (-1–126)	54 (0–154)	0 (0–60)	0.1932	0.3902
Other	0 (-1–83)	49 (-1–125)	0 (0–25)	0.6203	0.8778
Last medication date – last contact date (days), median (IQR)					
Completed study drug	1 (1–1)	1 (1–1)	-	0.1947	
Discontinued study drug					
Adverse events	197 (21–757)	1 (0–69)	54 (21–134)	<0.0001	0.0035
Major protocol deviation	4 (1–427)	21 (21–21)	807 (763–851)	0.2875	0.4385
Lost to follow-up	1 (0–99)	3 (1–450)	414 (414–414)	0.2034	0.8691
Voluntary withdrawal	34 (1–373)	112 (4–414)	863 (189–1091)	0.0084	0.0102
Other	15 (1–420)	1 (1–158)	160 (84–554)	0.0311	0.0242

P-value by chi-square test, Fisher's exact test, ANOVA, or Kruskal–Wallis test
†Adjusted P-value by chi-square test, Fisher's exact test with Bonferroni adjustment method, Dwass, Steel, Critchlow–Fligner multiple comparison, or Tukey's multiple comparison method.

Supplementary Figure 1. Cumulative Kaplan–Meier estimates of the time from febuxostat and allopurinol discontinuation to the first occurrence of a MACE (all study patients)

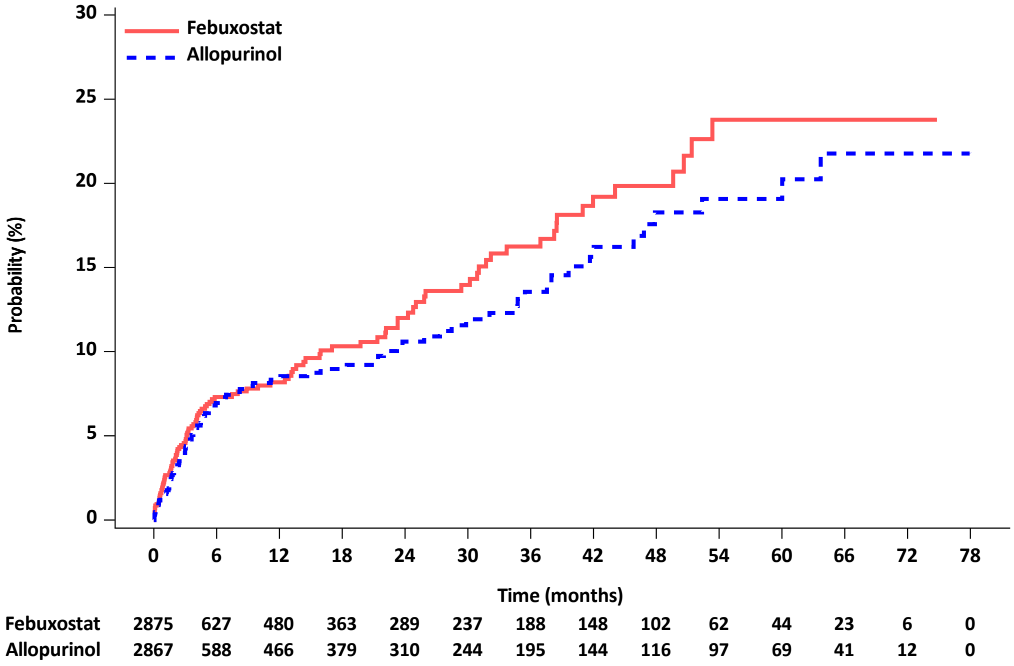

Supplementary Figure 2. Cumulative Kaplan–Meier estimates of the time to the first occurrence of an adjudicated MACE (during 6 months)

a. All study patients

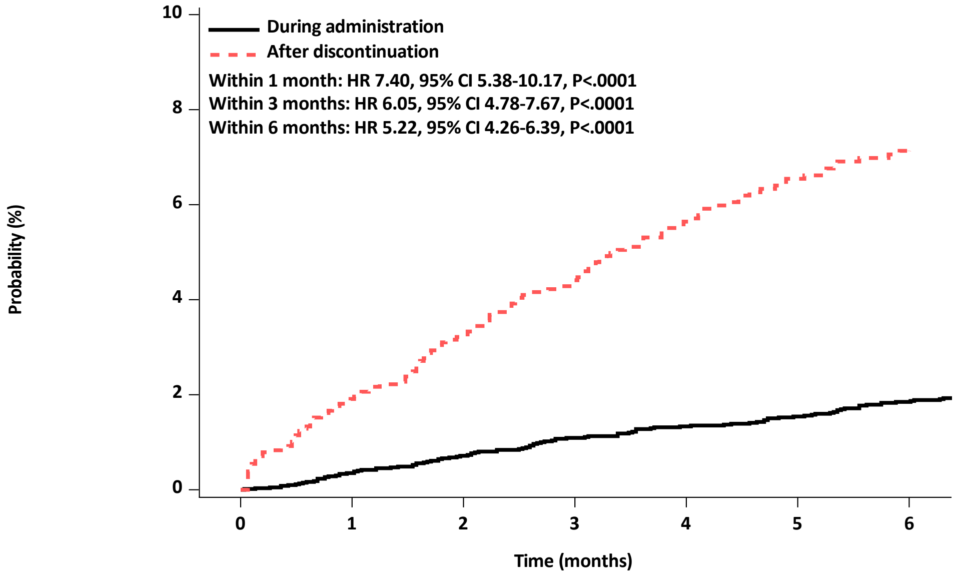

During administration	6190	5854	5617	5379	5254	5165	5001
After discontinuation	5742	1940	1691	1520	1401	1308	1215

b. Study patients with no abnormal clinical or vital signs during any of the study visits

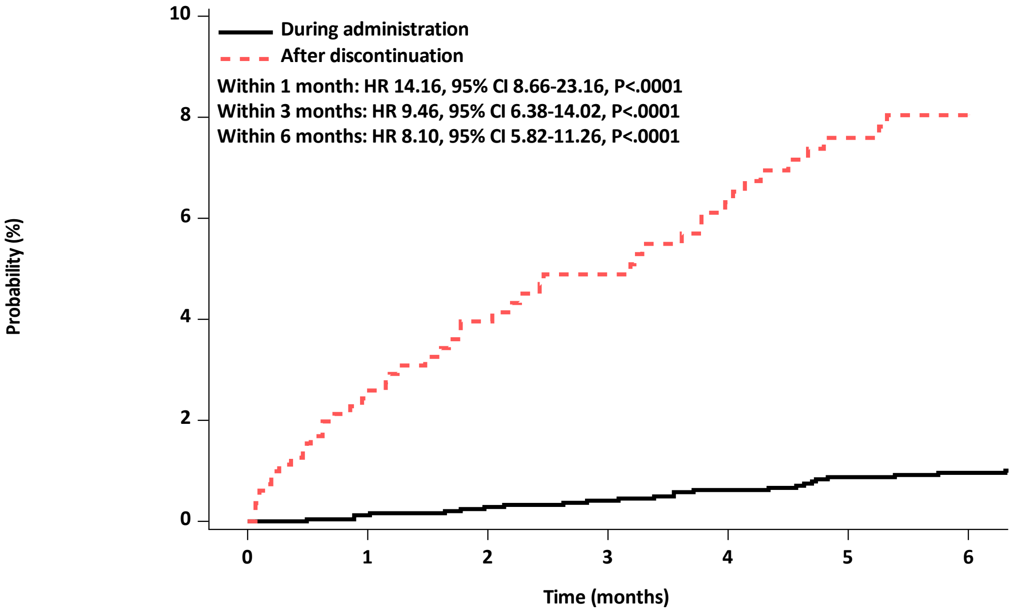

During administration	2517	2467	2416	2371	2346	2330	2297
After discontinuation	2379	617	533	482	449	424	388

Ghang B, *et al. RMD Open* 2022; 8:e001944. doi: 10.1136/rmdopen-2021-001944

c. Study patients who were followed up for more than 1 month after discontinuing the study drug (among the excluded patients; all patients with MACEs during administration and after discontinuation are included)

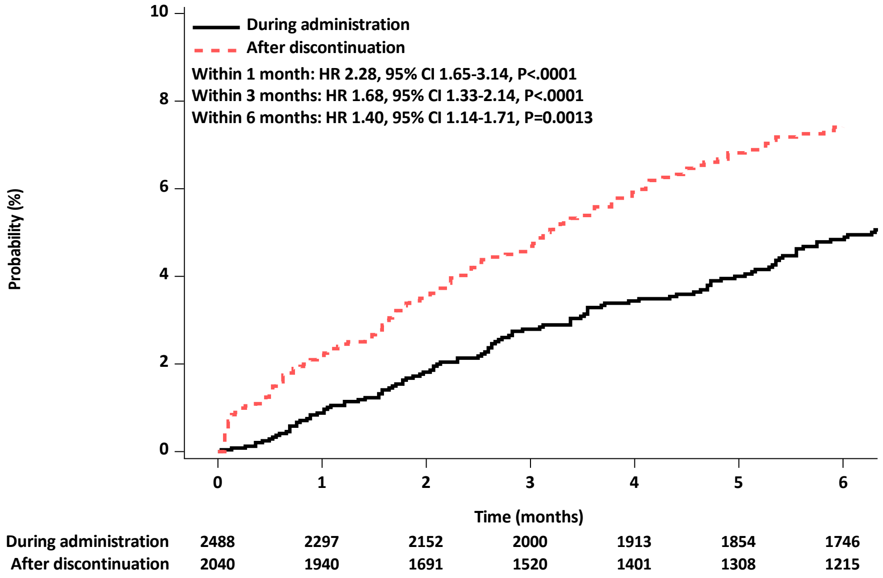

d. Study patients with no abnormal clinical or vital signs during the study visits (among the excluded patients; patients with MACEs during administration are only included in the 'during administration' group)

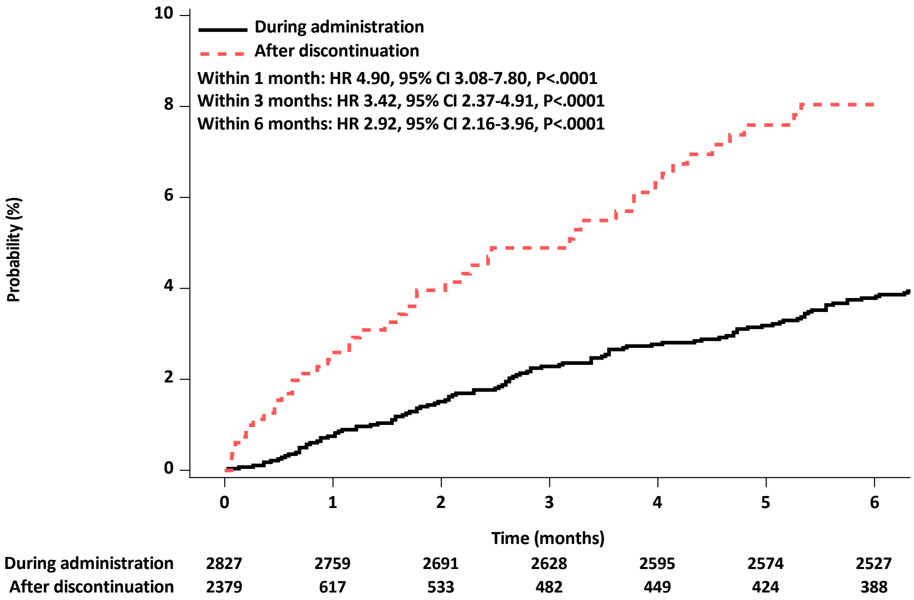

Ghang B, et al. RMD Open 2022; 8:e001944. doi: 10.1136/rmdopen-2021-001944

Supplementary Table 3. Comparative risk of MACEs between during administration and after discontinuation of the study drug (during 6 months; table of Supplementary Figure 2)

a. All study patients

Last study drug stop	No. of events	Person-years	Incidence rates per 100 person-years	IRR (95% CI)	P-value	HR (95% CI)	P-value
Within 1 month							
-During administration	448	14424	3.11 (2.83–3.41)	1 (Ref)		1 (Ref)	
-After discontinuation	44	190	23.13 (17.21–31.07)	7.45 (5.46–10.15)	<0.0001	7.40 (5.38–10.17)	<0.0001
Within 3 months							
-During administration	448	14424	3.11 (2.83–3.41)	1 (Ref)		1 (Ref)	
-After discontinuation	88	473	18.61 (15.10–22.94)	5.99 (4.77–7.53)	<0.0001	6.05 (4.78–7.67)	<0.0001
Within 6 months							
-During administration	448	14424	3.11 (2.83–3.41)	1 (Ref)		1 (Ref)	
-After discontinuation	128	812	15.77 (13.26–18.76)	5.08 (4.17–6.18)	<0.0001	5.22 (4.26–6.39)	<0.0001

Ghang B, *et al. RMD Open* 2022; 8:e001944. doi: 10.1136/rmdopen-2021-001944

b. Study patients with no abnormal clinical or vital signs during any of the study visits

Last study drug stop	No. of events	Person-years	Incidence rates per 100 person-years	IRR (95% CI)	P-value	HR (95% CI)	P-value
Total							
-During administration	138	6569	2.10 (1.78–2.48)	1 (Ref)		1 (Ref)	
-After discontinuation	64	942	6.79 (5.32–8.68)	3.23 (2.40–4.35)	<0.0001	3.01 (2.18–4.17)	<0.0001
Within 1 month							
-During administration	138	6569	2.10 (1.78–2.48)	1 (Ref)		1 (Ref)	
-After discontinuation	19	62	30.54 (19.48–47.87)	14.54 (9.00–23.48)	<0.0001	14.16 (8.66–23.16)	<0.0001
Within 3 months							
-During administration	138	6569	2.10 (1.78–2.48)	1 (Ref)		1 (Ref)	
-After discontinuation	32	151	21.17 (14.97–29.93)	10.07 (6.86–14.80)	<0.0001	9.46 (6.38–14.02)	<0.0001
Within 6 months							
-During administration	138	6569	2.10 (1.78–2.48)	1 (Ref)		1 (Ref)	
-After discontinuation	47	260	18.09 (13.59–24.08)	8.61 (6.18–11.99)	<0.0001	8.10 (5.82–11.26)	<0.0001

Ghang B, et al. RMD Open 2022; 8:e001944. doi: 10.1136/rmdopen-2021-001944

c. Study patients who were followed up for more than 1 month after discontinuing the study drug (among the excluded patients, all patients with MACEs during administration and after discontinuation are included)

Last study drug stop	No. of events	Person-years	Incidence rates per 100 person-years	IRR (95% CI)	P-value	HR (95% CI)	P-value
Total							
-During administration	448	3905	11.47 (10.46–12.59)	1 (Ref)		1 (Ref)	
-After discontinuation	208	3075	6.76 (5.91–7.75)	0.59 (0.50–0.70)	<0.0001	0.55 (0.45–0.66)	<0.0001
Within 1 month							
-During administration	448	3905	11.47 (10.46–12.59)	1 (Ref)		1 (Ref)	
-After discontinuation	44	164	26.79 (19.94–36.00)	2.33 (1.71–3.18)	<0.0001	2.28 (1.65–3.14)	<0.0001
Within 3 months							
-During administration	448	3905	11.47 (10.46–12.59)	1 (Ref)		1 (Ref)	
-After discontinuation	88	447	19.69 (15.98–24.27)	1.72 (1.37–2.16)	<0.0001	1.68 (1.33–2.14)	<0.0001
Within 6 months							
-During administration	448	3905	11.47 (10.46–12.59)	1 (Ref)		1 (Ref)	
-After discontinuation	128	786	16.29 (13.70–19.38)	1.42 (1.17–1.73)	0.0005	1.40 (1.14–1.71)	0.0013

Ghang B, *et al. RMD Open* 2022; 8:e001944. doi: 10.1136/rmdopen-2021-001944

d. Study patients with no abnormal clinical or vital signs during the study visits (among the excluded patients, patients with MACEs during administration are only included in the 'during administration' group)

Last study drug stop	No. of event	Person-years	Incidence rates per 100 person-years	IRR (95% CI)	P-value	HR (95% CI)	P-value
Total							
-During administration	448	7092	6.32 (5.76–6.93)	1(Ref)		1(Ref)	
-After discontinuation	64	942	6.79 (5.32–8.68)	1.08 (0.83–1.40)	0.5860	1.11 (0.84–1.48)	0.4626
Within 1 month							
-During administration	448	7092	6.32 (5.76–6.93)	1(Ref)		1(Ref)	
-After discontinuation	19	62	30.54 (19.48–47.87)	4.83 (3.05–7.65)	<0.0001	4.90 (3.08–7.80)	<0.0001
Within 3 months							
-During administration	448	7092	6.32 (5.76–6.93)	1(Ref)		1(Ref)	
-After discontinuation	32	151	21.17 (14.97–29.93)	3.35 (2.34–4.80)	<0.0001	3.42 (2.37–4.91)	<0.0001
Within 6 months							
-During administration	448	7092	6.32 (5.76–6.93)	1(Ref)		1(Ref)	
-After discontinuation	47	260	18.09 (13.59–24.08)	2.86 (2.12–3.87)	<0.0001	2.92 (2.16–3.96)	<0.0001

Supplementary Figure 3. Survival curves for MACE between during drug administration and after discontinuation under the assumption that participants who completed the trial did not have any MACE events.

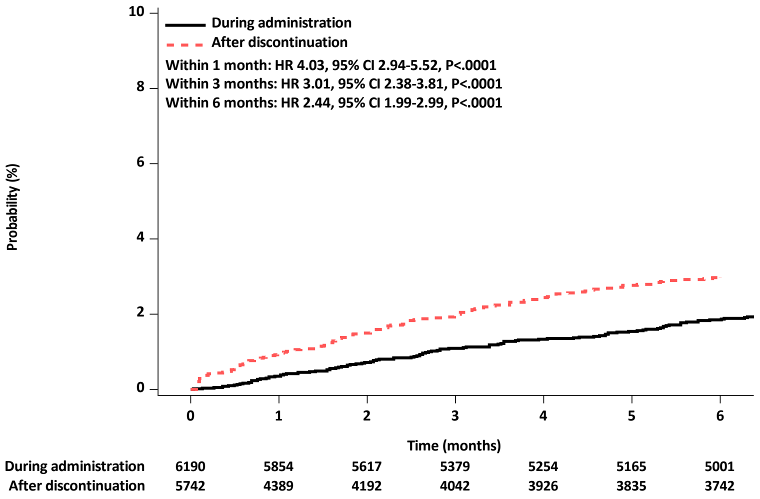

Supplementary Figure 4. Change in serum uric acid level based on the uric acid levels during and after drug administration

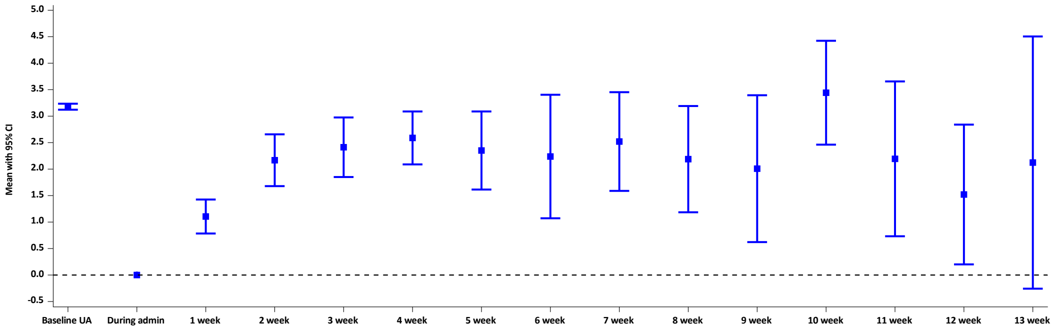

Supplementary Table 4. Baseline characteristics of patients with at least 1 year of drug administration according to MACE development after drug discontinuation after excluding patients with MACE during drug administration.

	CV event after discontinuation (n = 97)	No CV event after discontinuation (n = 3739)	P-value
Treatment, n(%)			0.2429
Febuxostat	54 (55.7)	1857 (49.7)	
Allopurinol	43 (44.3)	1882 (50.3)	
Age, Mean±SD	68.0 ± 9.1	64.5 ± 8.3	<.0001
Age≥65, n (%)	63 (64.9)	1833 (49.1)	0.0020
Male, n (%)	81 (83.5)	3168 (84.7)	0.7411
Median body weight (Interquartile range)	100.9 (87.0-113.4)	97.1 (84.4-112.3)	0.2032
Body mass index, Mean ± SD	35.0 ± 8.8	33.4 ± 6.8	0.0737
Race or ethnic group, n(%)			0.0070
White	81 (83.5)	2585 (69.1)	
Black or African American	11 (11.3)	629 (16.8)	
Others	5 (5.2)	525 (14.0)	
Smoker			0.1593
Never smoked	28 (28.9)	1437 (38.4)	
Current smoker	13 (13.4)	443 (11.8)	
Ex-smoker	56 (57.7)	1859 (49.7)	
Drink			0.7139
Never Drank	23 (23.7)	964 (25.8)	
Current Drinker	46 (47.4)	1829 (48.9)	
Ex-Drinker	28 (28.9)	946 (25.3)	
Baseline Glucose, Median (IQR)	118.0 (100.0-137.0)	109.0 (97.0-134.0)	0.0606
Baseline LDL	90.4 ± 37.8	88.2 ± 35.2	0.5479
Baseline SBP	134.8 ± 17.7	132.3 ± 16.8	0.1446
Baseline DBP	77.1 ± 12.0	77.8 ± 10.6	0.4946
Baseline Creatinine	1.38 ± 0.36	1.22 ± 0.32	<.0001
Stage of chronic kidney disease (eGFR)			<.0001
Stage 1 (90+)	4 (4.1)	445 (11.9)	
Stage 2a (75-89)	12 (12.4)	726 (19.4)	
Stage 2b (60-74)	17 (17.5)	1083 (29.0)	
Stage 3a (45-59)	29 (29.9)	948 (25.4)	
Stage 3b (30-44)	31 (32.0)	497 (13.3)	
Stage 4 (15-29)	4 (4.1)	38 (1.0)	
Baseline eGFR	55.0 ± 17.8	65.9 ± 18.6	<.0001
Baseline serum urate level, Mean±SD	9.4 ± 1.8	8.7 ± 1.6	<.0001
Follow-up Uric Acid	5.6 ± 1.7	5.5 ± 1.7	0.6800
Change from Baseline Uric Acid	3.8 ± 2.2	3.1 ± 2.1	0.0011
Baseline uroprotein			0.0902
Negative + Trace	79 (81.4)	3156 (84.9)	
+1	8 (8.2)	365 (9.8)	
+2 - +4	10 (10.3)	196 (5.3)	
Duration of gout, Median (IQR)	6.3 (2.1-19.0)	8.1 (3.1-18.0)	0.5454

Average sUA value for each subject collected from the end of the first year of treatment up-to 1 day after last dose date	5.5 ± 1.3	5.5 ± 1.4	0.7959
Median no. of gout flares (IQR)	1.0 (0.0-3.0)	1.0 (0.0-2.0)	0.0132
Presence of tophi, n(%)	25 (25.8)	799 (21.4)	0.2971
Cardiovascular risk factors and history, n(%)			
DM with small-vessel disease	37 (38.1)	1476 (39.5)	0.7911
Hypertension	95 (97.9)	3435 (91.9)	0.0294
Hyperlipidemia	84 (86.6)	3241 (86.7)	0.9811
MI	42 (43.3)	1360 (36.4)	0.1620
Hospitalization for unstable angina	29 (29.9)	969 (25.9)	0.3776
Coronary revascularization	48 (49.5)	1338 (35.8)	0.0056
Cerebral revascularization	4 (4.1)	70 (1.9)	1.0000
Congestive heart failure	32 (33.0)	645 (17.3)	<.0001
Stroke	14 (14.4)	480 (12.8)	0.6433
Peripheral vascular disease	20 (20.6)	419 (11.2)	0.0040
Initial Prophylactic Medication			0.3724
Colchicine 0.6 mg QD	86 (88.7)	3144 (84.1)	
Naproxen 250 mg BID + PPI	6 (6.2)	395 (10.6)	
Other + None	5 (5.2)	200 (5.3)	

Supplementary Table 5. Multivariable Cox regression analysis of MACE during administration in gout patients with more than 1 year of febuxostat or allopurinol administration

	Unadjusted		Adjusted (N=4169)	
	HR (95% CI)	P-value	HR (95% CI)	P-value
Treatment				
Allopurinol	1(Ref)		1(Ref)	
Febuxostat	1.04 (0.85-1.28)	0.7132	1.06 (0.86-1.31)	0.5658
Age (per 10 unit increase)	1.32 (1.16-1.49)	<.0001	1.17 (1.00-1.37)	0.0524
Male	1.14 (0.84-1.56)	0.3987	1.06 (0.76-1.48)	0.7447
Body mass index	1.00 (0.99-1.02)	0.6898	1.01 (0.99-1.02)	0.4475
Race or ethnic group				
White	1(Ref)		1(Ref)	
Black or African American	0.78 (0.58-1.06)	0.1096	0.87 (0.63-1.19)	0.3779
Others	0.37 (0.22-0.61)	<.0001	0.55 (0.33-0.92)	0.0219
Smoker				
Never smoked	1(Ref)		1(Ref)	
Current smoker	1.00 (0.70-1.43)	0.9889	0.94 (0.64-1.37)	0.7500
Ex-smoker	1.10 (0.88-1.38)	0.3833	0.91 (0.72-1.16)	0.4546
Drink				
Never Drank	1(Ref)		1(Ref)	
Current Drinker	0.95 (0.73-1.23)	0.6873	1.09 (0.82-1.44)	0.5492
Ex-Drinker	1.19 (0.90-1.59)	0.2198	1.21 (0.90-1.64)	0.2117
Baseline Glucose (per 10 unit increase)	1.03 (1.01-1.05)	0.0125	1.04 (1.01-1.06)	0.0060
Baseline LDL (per 10 unit increase)	1.04 (1.01-1.07)	0.0042	1.06 (1.03-1.09)	<.0001
Baseline SBP (per 10 unit increase)	1.10 (1.04-1.17)	0.0019	1.10 (1.02-1.19)	0.0099
Baseline DBP (per 10 unit increase)	0.99 (0.90-1.10)	0.8960	0.97 (0.85-1.10)	0.6188
**Change in sUA levels[a] (per 1 mg/dL increase)**	**1.03 (0.98-1.08)**	**0.3242**	**0.99 (0.95-1.04)**	**0.8115**
Stage of chronic kidney disease (eGFR)				
Stage 1 (90+)	1(Ref)		1(Ref)	
Stage 2a (75-89)	1.41 (0.84-2.36)	0.1956	1.24 (0.74-2.09)	0.4187
Stage 2b (60-74)	1.64 (1.01-2.65)	0.0459	1.26 (0.77-2.08)	0.3528
Stage 3a (45-59)	2.05 (1.27-3.31)	0.0032	1.39 (0.84-2.31)	0.1956
Stage 3b (30-44)	2.85 (1.74-4.66)	<.0001	1.80 (1.06-3.08)	0.0308
Stage 4 (15-29)	3.08 (1.30-7.29)	0.0104	1.86 (0.75-4.60)	0.1788
Baseline uroprotein				
Negative + Trace	1(Ref)		1(Ref)	
+1	1.52 (1.12-2.06)	0.0069	1.29 (0.94-1.76)	0.1136
+2 - +4	1.46 (0.95-2.23)	0.0814	1.06 (0.68-1.67)	0.7937
Duration of gout (per 10 unit increase)	1.09 (1.00-1.18)	0.0570	1.07 (0.98-1.17)	0.1066
Presence of tophi	1.05 (0.82-1.35)	0.6865	1.06 (0.81-1.37)	0.6884
Cardiovascular risk factors and history				
DM with small-vessel disease	0.81 (0.65-1.01)	0.0639	0.83 (0.64-1.07)	0.1526
Hypertension	1.46 (0.92-2.31)	0.1102	0.96 (0.60-1.55)	0.8747
Hyperlipidemia	1.46 (1.00-2.12)	0.0477	0.98 (0.67-1.45)	0.9342
Myocardial infarction	1.89 (1.53-2.32)	<.0001	1.51 (1.20-1.89)	0.0004
Hospitalization for unstable angina	1.75 (1.42-2.17)	<.0001	1.44 (1.15-1.81)	0.0014

Coronary revascularization	1.99 (1.61-2.45)	<.0001	1.46 (1.15-1.85)	0.0021
Cerebral revascularization	1.73 (1.00-3.01)	0.0519	1.35 (0.77-2.38)	0.2991
Congestive heart failure	2.21 (1.77-2.75)	<.0001	1.49 (1.16-1.90)	0.0015
Stroke	1.77 (1.38-2.28)	<.0001	1.71 (1.32-2.23)	<.0001
Peripheral vascular disease	1.68 (1.28-2.21)	0.0002	1.51 (1.12-2.03)	0.0061
Initial Prophylactic Medication				
Colchicine 0.6 mg QD	1(Ref)		1(Ref)	
Naproxen 250 mg BID + PPI	0.74 (0.51-1.08)	0.1209	0.84 (0.57-1.24)	0.3800
Other + None	0.54 (0.29-1.02)	0.0571	0.52 (0.27-0.98)	0.0425

[a]Change in sUA levels: determined the calculating the difference between baseline and last measured serum uric acid prior to drug discontinuation
DBP: diastolic blood pressure, DM: Diabetes mellitus, IQR: Interquartile range, LDL: low density lipoprotein, SBP: systolic blood pressure, sUA: serum uric acid.

Supplementary Figure 5. Time to death after last medication

a. All-cause death

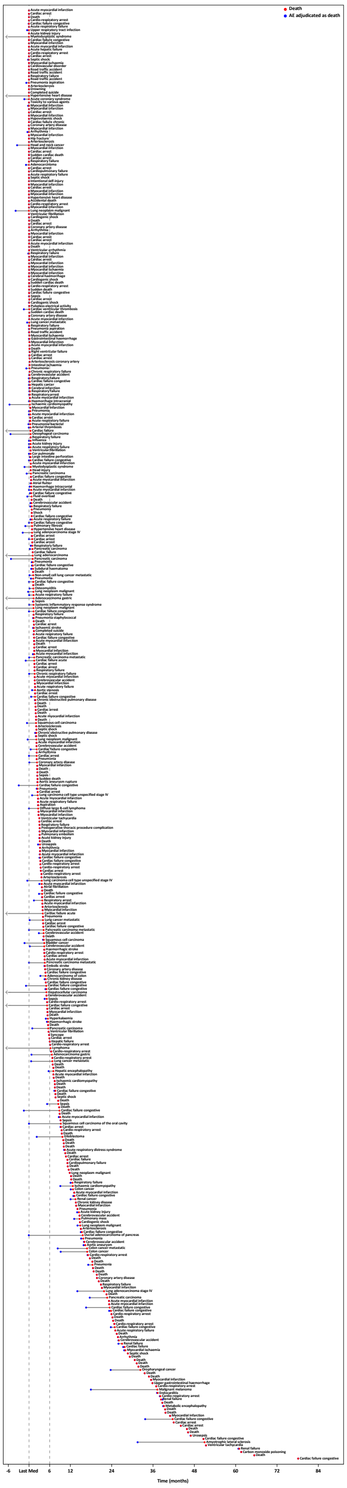

b. CV death (during 6 months)

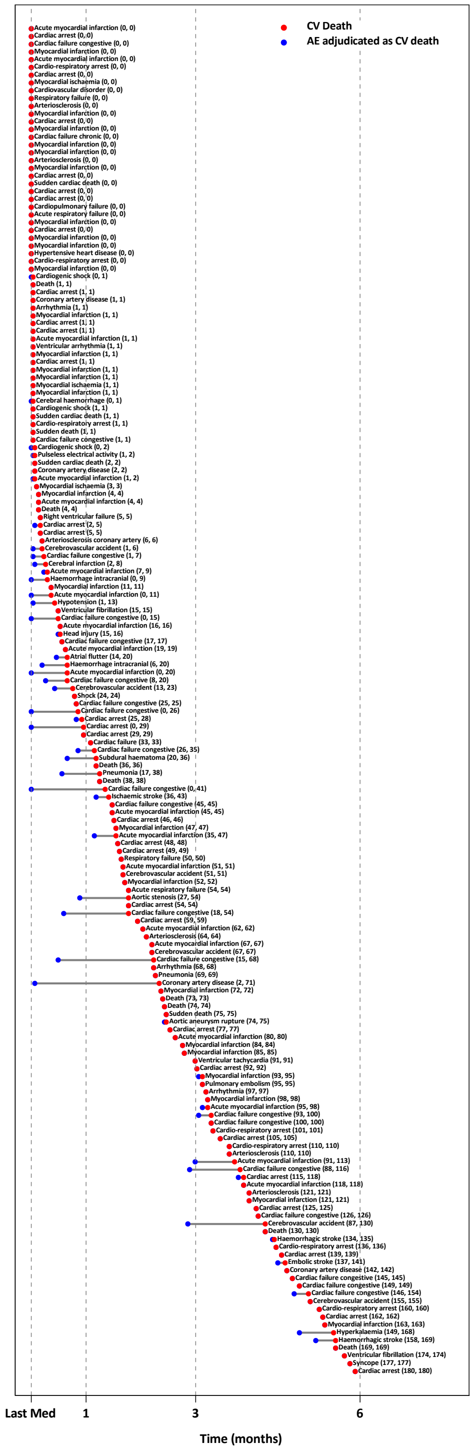

c. Non-CV death (during 6 months)

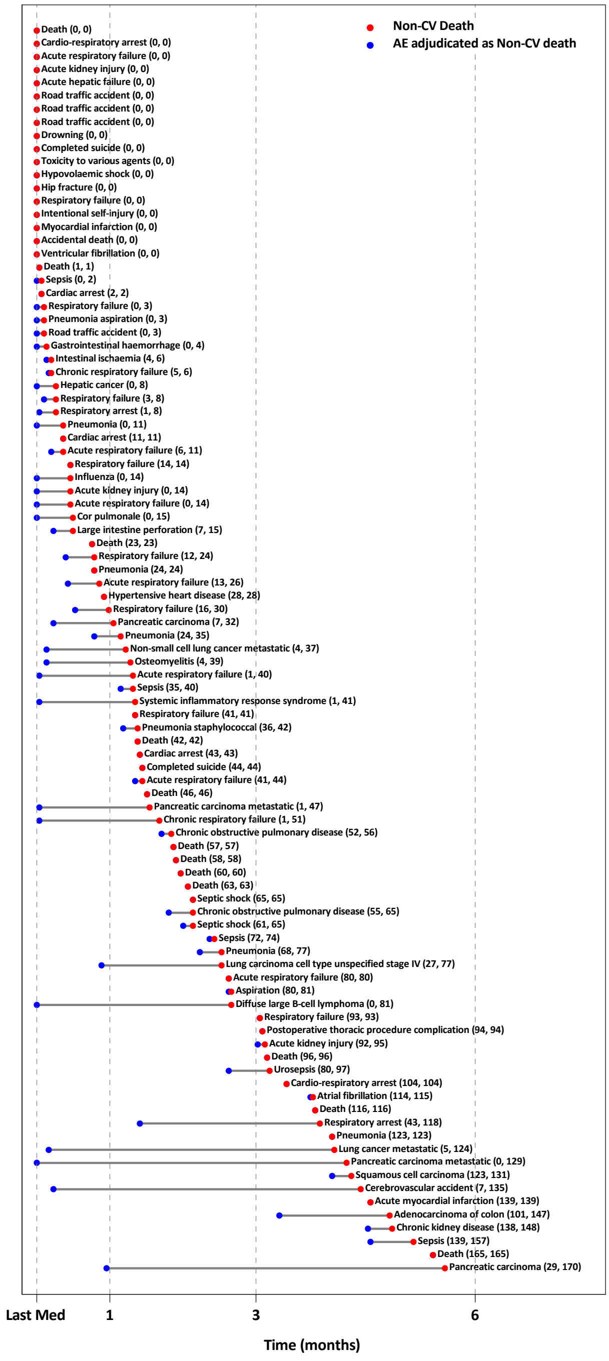

20

Supplementary Table 6. Comparative mortality between cardiovascular vs non-cardiovascular death (all study patients)

	Total (n = 442)	CV death (n = 234)	Non-CV death (n = 208)	P-value
Death within 1 month, n (%)				0.0014
No	278 (62.9)	131 (56.0)	147 (70.7)	
Yes	164 (37.1)	103 (44.0)	61 (29.3)	
Death within 3 months, n (%)				0.0005
No	195 (44.1)	85 (36.3)	110 (52.9)	
Yes	247 (55.9)	149 (63.7)	98 (47.1)	
Death within 6 months, n (%)				<0.0001
No	128 (29.0)	42 (17.9)	86 (41.3)	
Yes	314 (71.0)	192 (82.1)	122 (58.7)	

Supplementary Table 7. Comparative mortality between cardiovascular vs non-cardiovascular death in patients with no abnormal clinical or vital signs during the study visits (excluding death induced by AEs, which occurred up to 1 day after last medication)

	Total (n = 123)	CV death (n = 63)	Non-CV death (n = 60)	P-value
Death within 1 month, n (%)				0.0191
No	111 (90.2)	53 (84.1)	58 (96.7)	
Yes	12 (9.8)	10 (15.9)	2 (3.3)	
Death within 3 months, n (%)				0.0470
No	86 (69.9)	39 (61.9)	47 (78.3)	
Yes	37 (30.1)	24 (38.1)	13 (21.7)	
Death within 6 months, n (%)				0.0014
No	68 (55.3)	26 (41.3)	42 (70.0)	
Yes	55 (44.7)	37 (58.7)	18 (30.0)	

Ghang B, *et al. RMD Open* 2022; 8:e001944. doi: 10.1136/rmdopen-2021-001944

Supplementary Figure 6. Adverse events that occurred from 2 days after the last medication, adjudicated as death in patients with no abnormal clinical or vital signs during the study visits

a. Adverse events adjudicated as all-cause death

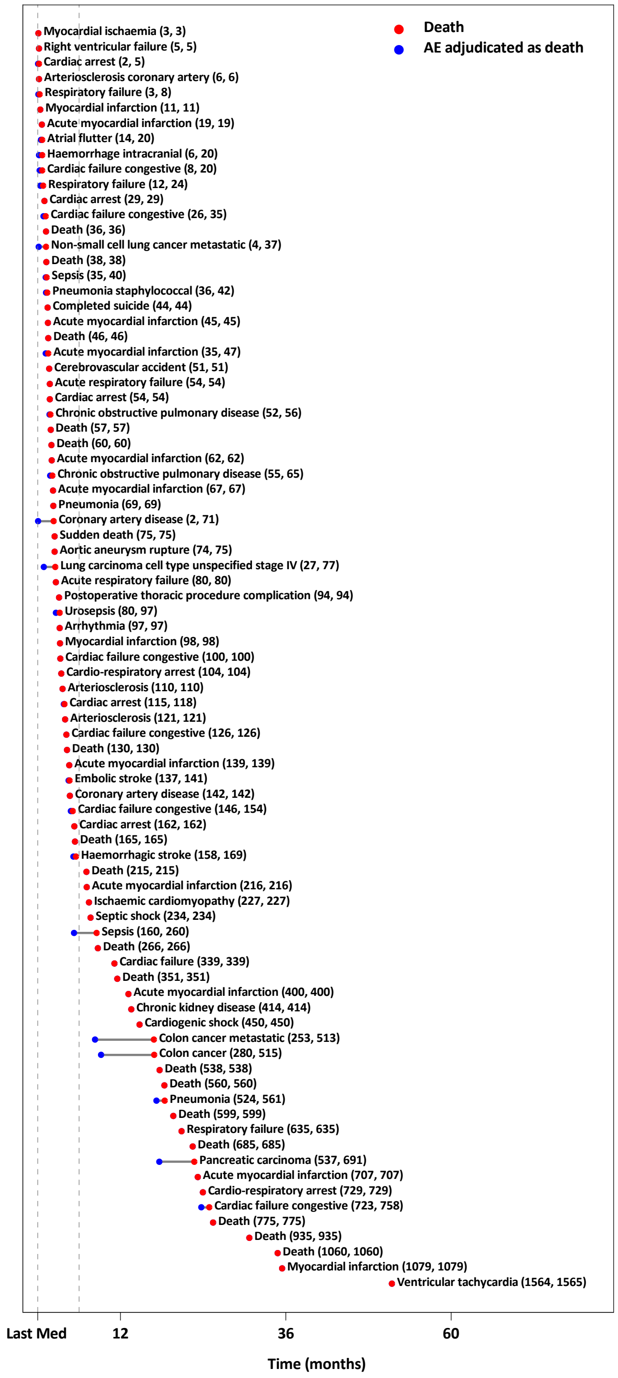

Ghang B, *et al. RMD Open* 2022; 8:e001944. doi: 10.1136/rmdopen-2021-001944

a. Adverse events adjudicated as cardiovascular death for 6 months

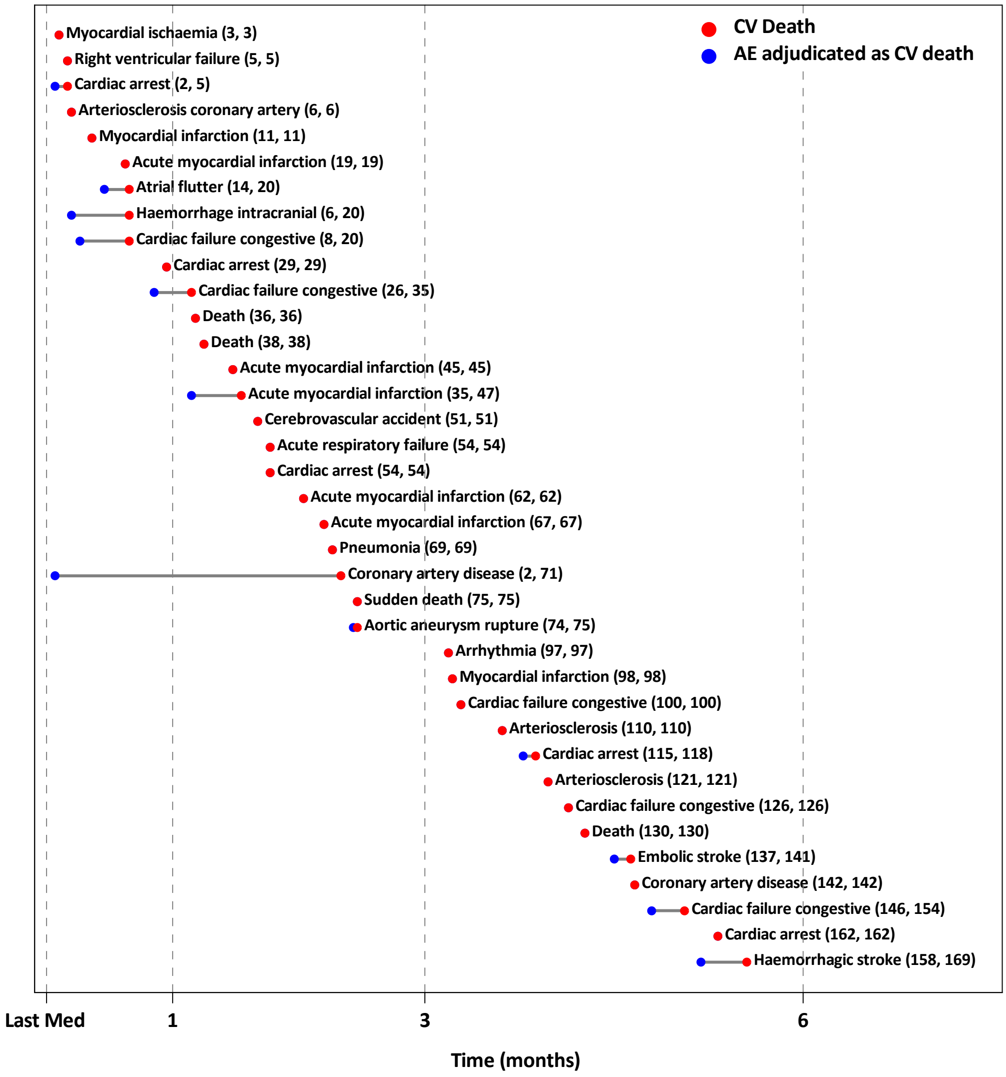

b. Adverse events adjudicated as non-cardiovascular death for 6 months

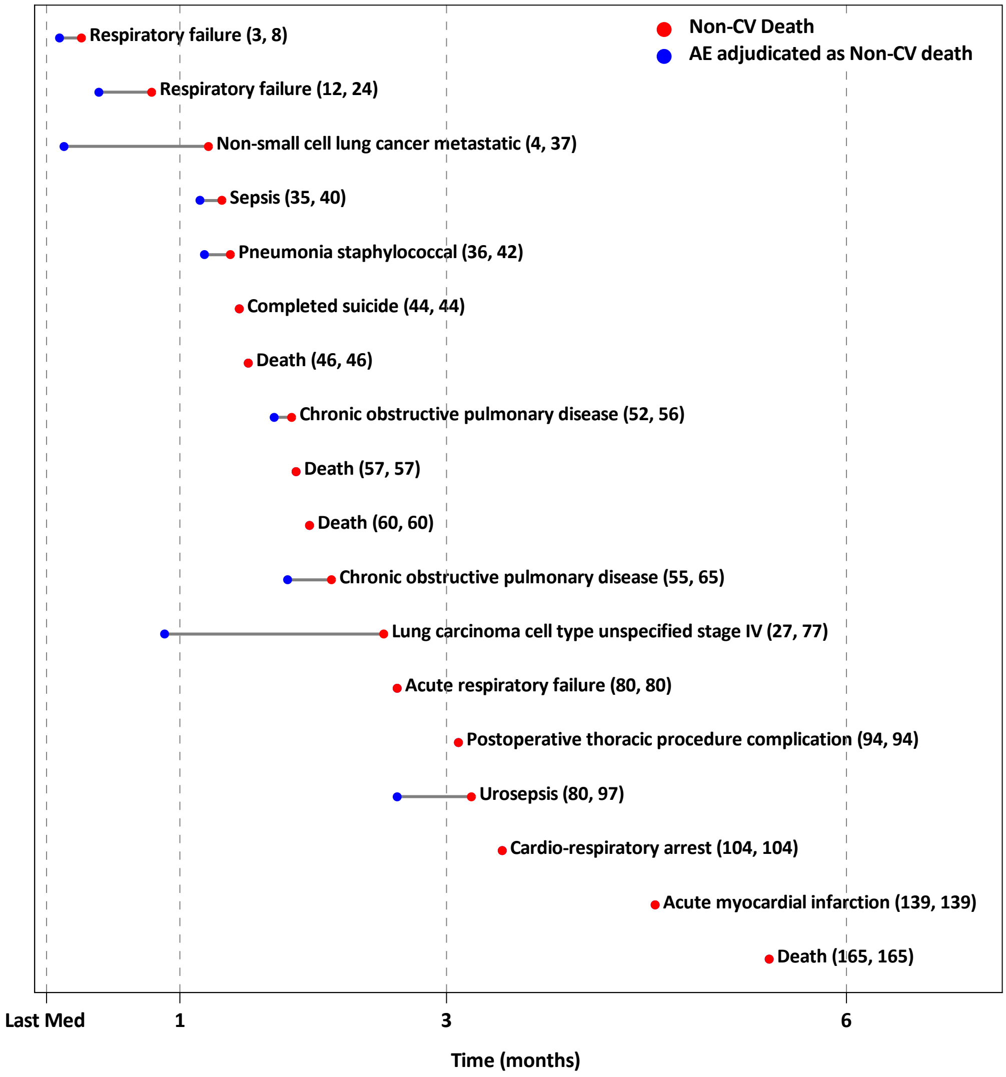

c. Cumulative Kaplan–Meier estimates of the time to adverse events adjudicated as death

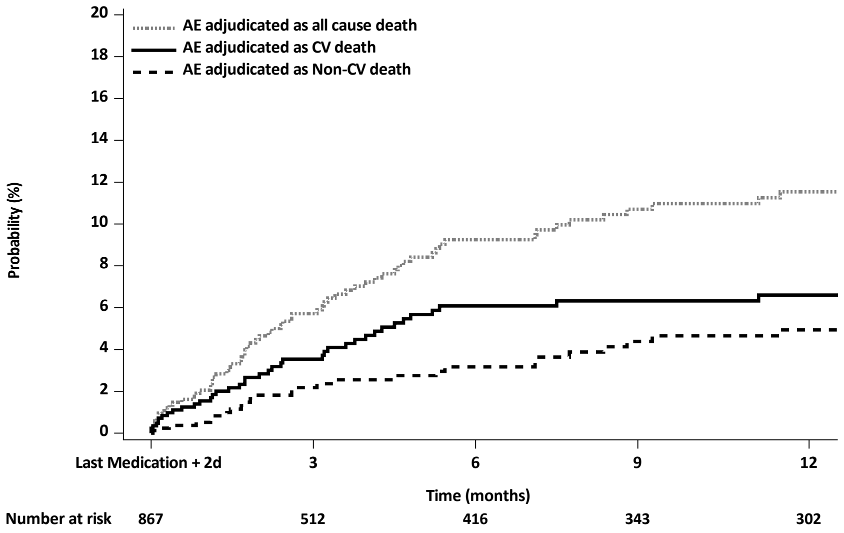

Supplementary Table 8. Comparative incidence of adverse events that occurred from 2 days after the last medication, adjudicated as between cardiovascular vs non-cardiovascular death in patients with no abnormal clinical or vital signs during the study visits.

	Total (n = 123)	CV death (n = 63)	Non-CV death (n = 60)	P-value
Death within 1 month, n (%)				0.0413
No	107 (87.0)	51 (81.0)	56 (93.3)	
Yes	16 (13.0)	12 (19.0)	4 (6.7)	
Death within 3 months, n (%)				0.0765
No	85 (69.1)	39 (61.9)	46 (76.7)	
Yes	38 (30.9)	24 (38.1)	14 (23.3)	
Death within 6 months, n (%)				0.0026
No	67 (54.5)	26 (41.3)	41 (68.3)	
Yes	56 (45.5)	37 (58.7)	19 (31.7)	

Ghang B, *et al. RMD Open* 2022; 8:e001944. doi: 10.1136/rmdopen-2021-001944
